# The cysteine-rich domain of TET2 binds preferentially to mono- and dimethylated histone H3K36

**DOI:** 10.1093/jb/mvx004

**Published:** 2017-01-27

**Authors:** Kazuyuki Yamagata, Akira Kobayashi

**Affiliations:** 1 Division of Newborn Medicine, Children's Hospital Boston; 2 Department of Cell Biology, Harvard Medical School, 300 Longwood Avenue, Boston, MA 02115, USA; 3 Organization for Research Initiatives and Development, Doshisha University; 4 Laboratory for Genetic Code, Graduate School of Life and Medical Sciences, Doshisha University, 1-3 Tatara Miyakodani, Kyotanabe 610-0394, Japan

**Keywords:** epigenetics, TET2, leukaemia, histone H3K36 methylation, histones < chromosomes

## Abstract

Missense mutations in *Ten-eleven translocation 2* (*TET2*) gene are frequently found in leukaemia patients. Although mutations span the entire coding region, they tend to cluster in the C-terminal enzymatic domain and a cysteine-rich (CR) domain of unknown function. Herein, we found the CR domain binds chromatin preferentially at the histone H3 tail by recognising H3 lysine 36 mono- and dimethylation (H3K36me1/2). Importantly, missense mutations in the CR domain perturbed TET2 recruitment to the target locus and its enzymatic activities. Our findings identify a novel H3K36me recognition domain and uncover a critical link between histone modification and DNA hydroxylation in leukaemogenesis.

Myeloid leukaemia is a type of blood cancer in which normal myeloid differentiation is impaired. Multiple gene mutations that contribute to cancer initiation and/or progression have been identified in myeloid leukaemia patients, among which missense mutations in the ten-eleven translocation 2 (TET2) oncogene, a member of the tet family, have been found in several independent patient cohorts with massive myeloproliferative disorders (1). Consistent with the idea that TET2 may play a role in leukaemia, Tet2 mutant mice show increased hematopoietic stem cell self-renewal and myeloid transformation (2). However, the mechanism by which TET2 mutations cause myeloid leukaemia is unclear. TET1, an ortholog of TET2, catalyses the conversion of 5-methylcytosine (5mC) in DNA to 5-hydroxymethylcytosine (hmC) (3), which suggests that epigenetic regulation plays a critical role in leukaemia development caused by TET2 mutations. Recent studies reveal that TET2 mutations occur throughout the CDS of TET2 in various leukaemia patients. Importantly, however, approximately ∼15% of missense mutations are clustered in a cysteine-rich (CR) domain (CR_TET2_) (1) of unknown function.

ATR-X (alpha-thalassemia/mental retardation, X-linked) syndrome is a human congenital disorder that causes severe intellectual disability. We previously reported that mutations in ADD_ATRX_, which are responsible for ATR-X, are associated with impaired localization of pericentromeric heterochromatin (4). Thus, we hypothesized that the CR domain might play a role in the association between TET2 and chromatin, and predicted that mutations in the CR domain associated with cancer might affect chromatin interactions. To test this possibility, we examined the subnuclear distribution of HA-tagged wild-type (WT) and mutant TET2 (Fig. 1A) in HEK293T cells by biochemical fractionation. We transfected WT or patient-derived TET2 mutants into HEK293T cells and prepared whole-cell extracts and cell fractions, and determined the distribution of TET2 by direct immunoblotting using anti-HA antibody (Fig. 1B and see online supplementary material for a colour version of Fig. S1). TET2 WT was present almost exclusively in the chromatin-enriched fraction but barely detectable in the cytosolic or soluble nuclear fractions (Fig. 1B, Panel 1). CR mutants (Panels 2 and 3) but not the double-stranded β-helix (DSBH) domain mutant (Panel 4) were only weakly detectable in the chromatin-enriched fraction, suggesting that the CR domain recognizes chromatin and its mutation decreases its binding to chromatin.

**Fig. 1:**
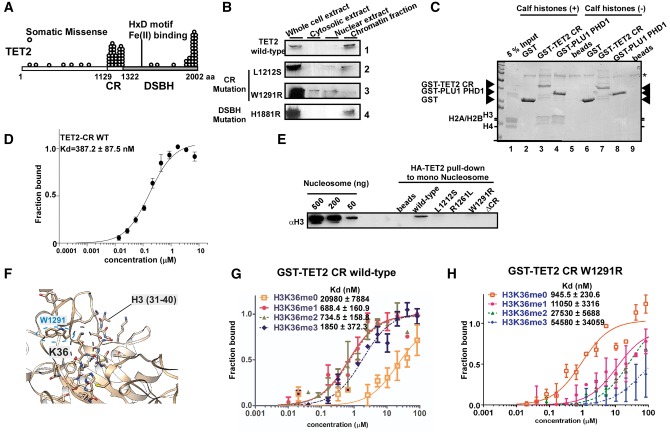
**The CR domain of TET2 is a nucleosome-binding module and preferentially recognises mono- and demethylated H3K36 peptides**. (**A**) TET2 mutations (circles) are predominantly clustered in either the cysteine-rich (CR, aa 1129 − 1322) or double-stranded β-helix domain (DSBH, aa 1323 − 2002). aa, amino acid. (**B**) Wild-type and DSBH TET2 domain mutants are enriched in the chromatin fraction, unlike CR domain mutants. HEK293T cells were transfected with the indicated expression plasmids and subjected to biochemical fractionation. Cell-equivalent fractions, whole-cell extracts, soluble cytosolic fractions, soluble nuclear fractions and chromatin fractions were probed by immunoblotting with anti-HA antibody. (**C**) The CR region preferentially binds histone H3. GST pull-down assays were performed using calf histones with GST, GST-TET2 CR or GST-PLU1 PHD1. Total amounts are shown following Coomassie brilliant blue (CBB) staining. The asterisk indicates BSA. (**D**) Titration curves and binding constants for mononucleosome binding to the wild-type CR domain. The Kd was determined by Monolith NT.115 and subsequently analysed by PRISM software. (**E**) Full-length TET2 CR mutations severely affect mononucleosome binding *in vitro*. Recombinant TET2 (50 ng) was incubated with 50 ng of mononucleosomes, and binding was determined by western blotting. (**F**) Binding model of TET2 (pdb: 5DEU) with H3 (31 − 40) peptides generated using CABS-dock. The H3 peptide is shown in stick representation, and TET2 (5DEU) is shown in ribbon representation. (**G** and **H**) Experimental microscale thermophoresis-derived binding affinities for unmodified and K36me peptide binding to GST-TET2 CR wild-type (G) and W1291R (H). Kd values were determined by Monolith NT.115 and subsequently analysed by PRISM software.

To determine whether TET2 also binds histones via its CR domain, CR_TET2_ (residues 1129 − 1322) was fused to glutathione S-transferase (GST) and incubated with calf histones. GST-PLU1 PHD1 was used as a positive control (Fig. 1C, Lane 4). We detected binding between CR and the histones, with H3 as the preferred histone (Fig. 1C, Lane 3). The Kd of the binding between CR_TET2_ and nucleosomes was determined by microscale thermophoresis (5) and found to be 387.2 ± 87.5 nM (WT) (Fig. 1D). We then determined whether compromised H3 binding is a general consequence of mutations associated with leukaemia. Full-length recombinant TET2 (WT; 50 ng) bound to ∼50 ng of mononucleosome but CR mutants did not (Fig. 1E), indicating that CR_TET2_ is important for nucleosome recognition. We, therefore, concluded that CR_TET2_ is a nucleosome-binding module and mutations in this domain can affect its binding.

The PWWP domain of DNA methyltransferases assists in CpG methylation by recognising H3K36me3 (6). Thus, we wondered whether the CR_TET2_ domain would assist in TET2 DNA demethylation by binding to methylated lysine residues in the nucleosome. To test this hypothesis, recombinant GST-fused CR domain was prepared and incubated with biotinylated histone H3 peptides methylated at various lysine residues by microscale thermophoresis. CR_TET2_ showed preferential binding to histone H3K36me1/2 (see online supplementary material for Table). We then simulated the binding mechanisms between H3 peptides and TET2 using CABS-dock (7). Interestingly, H3 (31 − 40) peptides appeared to interact with the DNA recognition pocket of CR_TET2_ (see online supplementary material for a colour version of Fig. S2) (8), which is frequently mutated in leukaemia patients (1). In addition, W1291 was predicted to lie close to H3K36 (Fig. 1F). Because the W1291R mutation is often found in leukaemia patents (1), we next tested the importance of the sequence surrounding H3 in histone binding to WT and W1291R mutant proteins. Histone H3_21-44_ K36me1 (688.4 ± 160.9 nM) and K36me2 peptides (734.5 ± 158.8 nM) showed similar binding affinity (Fig. 1H), whereas binding was impaired for unmodified H3_21-44_ (20,980 ± 7,884 nM) peptides. On the other hand, the W1291R mutant displayed a significantly reduced binding affinity to histone H3_21-44_ K36me1 (11,050 ± 3,316 nM) and K36me2 peptides (27,530 ± 5,688 nM); Fig. 1H), suggesting that W1291 is an important residue for recognition of H3K36 methylation. Surprisingly, the W1291R mutant bound preferentially to histone H3_21-44_ K36me0 peptide (945.5 ± 230.6 nM), indicating that CR mutations can affect the recognition of K36 methylation. Collectively, these results showed that CR_TET2_ binds preferentially methylated H3K36 and suggest that mutations in the CR domain of leukaemia patients may compromise its recognition ability *in vitro*.

Next, we investigated whether H3K36 methylation coincides with TET2 localization *in vivo*. Because the full-length TET2 protein bound preferentially to dimethylated histone H3K36 *in vitro* (data not shown), we examined the subnuclear distribution of HA-tagged WT and mutant TET2 in HeLa cells after anti-H3K36me2 antibody staining by immunofluorescence and confocal microscopy. TET2 WT and the DSBH mutant were predominantly localized to the nucleus (Fig. 2A and D, HA, left panel), and line profile analysis revealed that both were colocalized with H3K36me2 (right panel). CR_TET2_ mutants were also localized to the nucleus (Fig. 2B and C, HA, left panel), but neither colocalized well with the H3K36me2 signal (right panel). Next, we validated TET2 recruitment to chromatin by ChIP-qPCR. The results showed that the TET2 R1214W mutant was unable to recruit either TET2-bound regions (Fig. 2E and see online supplementary material for a colour version of Fig. S3A) or TET2-unbound regions (Fig. 2F and see online supplementary material for a colour version of Fig. S3B). By contrast, the W1291R mutant that bound preferentially to H3K36me0 (Fig. 1H) was abnormally recruited at both target and non-target loci (Fig. 2E,F and see online supplementary material for a colour version of Fig. S3). Taken together, the results suggest that mutations in the CR domain of TET2 disrupt recognition of H3K36 methylation, which causes mis-localization to the genomic locus and incorrect recruitment.

**Fig. 2:**
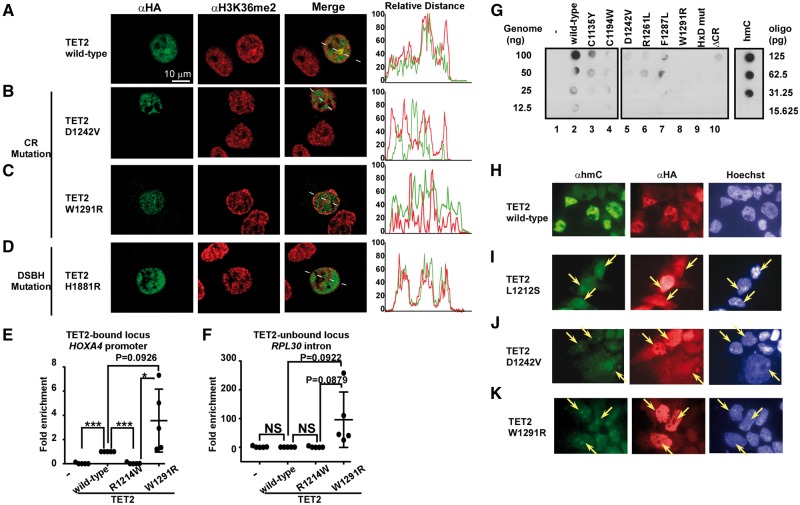
**TET2 CR domain mutations disrupt the recognition of H3K36 methylation, its cellular localization, and enzyme activity *in vivo***. (**A–D**) Wild-type TET2 (A) and the DSBH domain mutant (H1881R) (D) but not CR domain mutants (B and C) localize to H3K36me2 loci. HeLa cells were transfected with the indicated expression plasmids and analysed by immunostaining using confocal fluorescence microscopy. Line profile analysis of colocalization between TET2 proteins and H3K36me2 is shown in the panel on the right. The white bar indicates 10 μm. (**E** and **F**) CR mutations disrupt the correct localization of TET2-bound (E) and TET2-unbound (F) loci. HEK293T cells were transfected with the indicated expression plasmids and analysed by ChIP-qPCR. *P<0.05; ***P<0.0001 (student *t*-test). (**G**) TET2 mutations in the CR domain alter enzymatic activity in cells. Dot-blot analysis of 5-hmC levels in HEK293T cells over-expressing wild-type or mutant TET2. Oligonucleotides containing hmC or DNA from HEK293T cells transfected with TET2 containing mutations in the HxD motif (TET2-HxDmut) were used for positive and negative controls, respectively. (**H**–**K**). Immunocytochemical detection of 5-hmC (green) and HA-TET2 (red) in HEK293T cells transiently transfected with wild-type or mutant TET2. Arrows indicate signature nuclear dot patterns and euchromatin regions.

Finally, we investigated whether mutations in the CR domain of TET2 also affect its cellular catalytic activity *in vivo*. All patient-derived CR mutations tested displayed low or no catalytic activity in cells (Fig. 2G, Lanes 3 − 8). Some mutants also showed altered expression levels (see online supplementary material for a colour version of Fig. S4A, HA), but the enzyme activity was low regardless of the expression level (see online supplementary material for a colour version of this Fig. S4B and C). We further confirmed the subnuclear distribution of HA-tagged WT and mutant TET2 in HEK293T cells by immunofluorescence microscopy to determine the role of CR_TET2_ in nuclear localization and enzyme activity of TET2. HA-tagged TET2 (HA-TET2) showed clear nuclear localization that coincided with the level of the 5hmC signal (Fig. 2H). However, CR_TET2_ mutants were diffusely localized to the nucleus, and some mutants formed abnormal foci (Fig. 2I–K and see online supplementary material for a colour version of Fig. S5A–D). Consistent with the results in Fig. 2G, CR_TET2_ mutants exhibited a reduced hmC signal (Fig. 2I–K and see online supplementary material for a colour version of Fig. S5) and some also formed foci (arrows in Fig. 4I–K and see online supplementary material for a colour version of Fig. S5A–E), suggesting that colocalization with HA-TET2 did not occur. These results suggest that CR_TET2_ mutations abolish both enzymatic activity and cellular localization. Together, our findings suggest that leukaemia-derived mutations of CR_TET2_ impair catalytic activity in cells.

In this report, we demonstrated that CR_TET2_ binds preferentially to mono- and dimethylated H3K36, and leukaemia-related mutations disrupt binding to the target genome (see online supplementary material for a colour version of Fig. S6). Notably, the binding of the W1291R mutant to non-TET2 loci is the first report that mutations in CR_TET2_ can change its genomic localization, potentially opening the prospect that genomic mis-localization of CR_TET2_ mutants to non-target loci could serve as a biomarker for leukaemia. Loss of TET also contributes to cancer development in many cancers through abnormal regulation of DNA methylation (9), especially in leukaemias (1). Given that abnormal DNA methylation occurs in cancer and TET2 mutations are found in elderly people not showing signs of leukaemia, our findings indicate that CR_TET2_ mutations likely cause mis-localization of mutated TET2 proteins, resulting in abnormal DNA methylation and gene expression during the early stages of leukaemogenesis.

In conclusion, our results reveal an important relationship between TET2 and H3K36 methylation. The discovery of their direct interaction via the CR domain sheds new light on the connection between DNA hydroxylation and histone methylation, and its impact on chromatin and DNA biology, the pathogenic mechanisms of TET2-mutated leukaemia, and the epigenetic mechanisms in which they participate.

## Supplementary Data

Supplementary Data are available at *JB* online.

## Supplementary Material

Supplementary Data
