# Supplementary figures and images for: The cysteine-rich domain of TET2 binds preferentially to mono- and dimethylated histone H3K36
Source: J Biochem. 2017 Jan 27;161(4):327–30. doi: 10.1093/jb/mvx004 (PMC5412023; doi:10.1093/jb/mvx004)

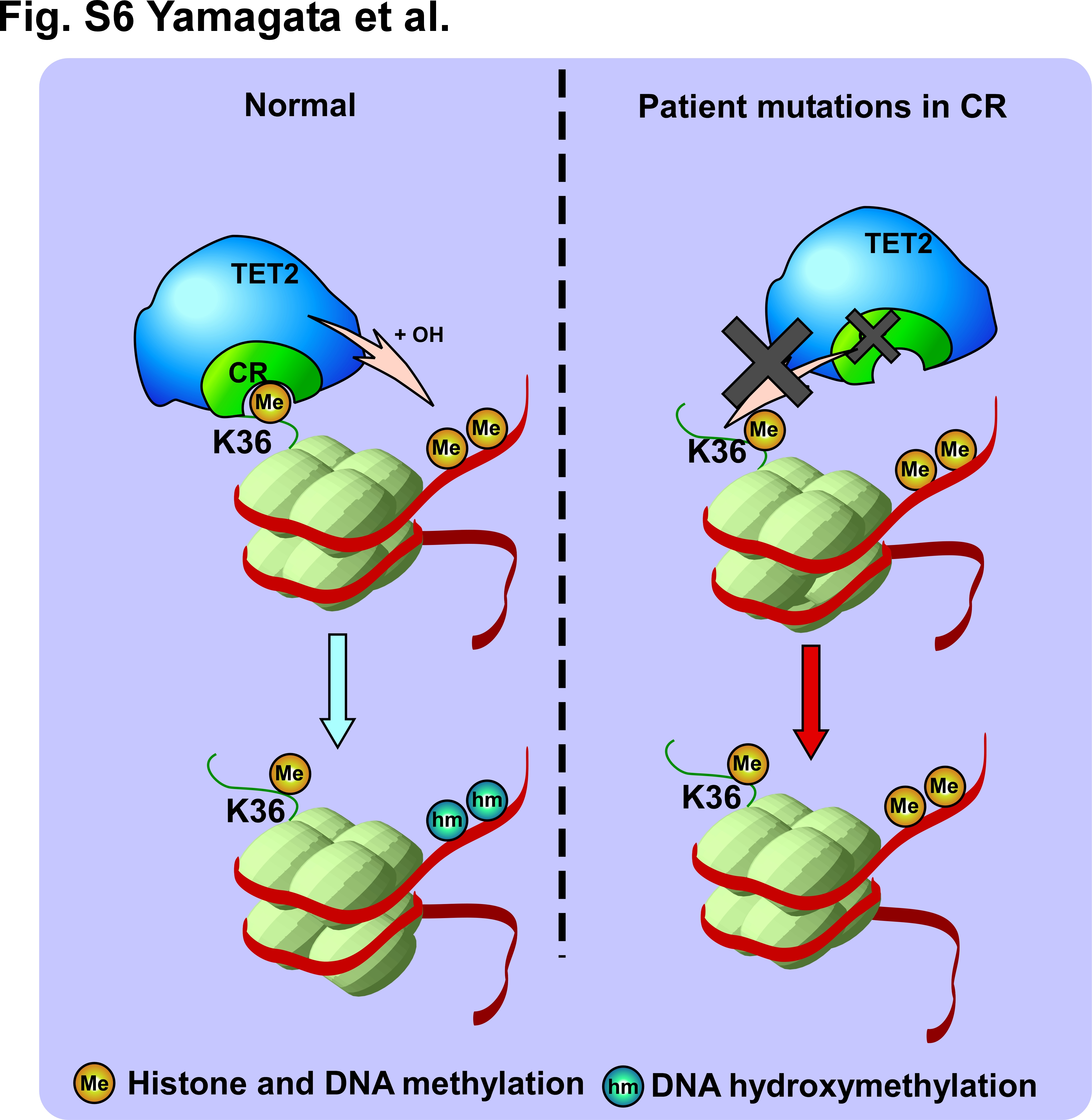

Supplement: Supplementary Data [file mvx004_Supp.zip › jb-17-01-0002-File010.jpg]

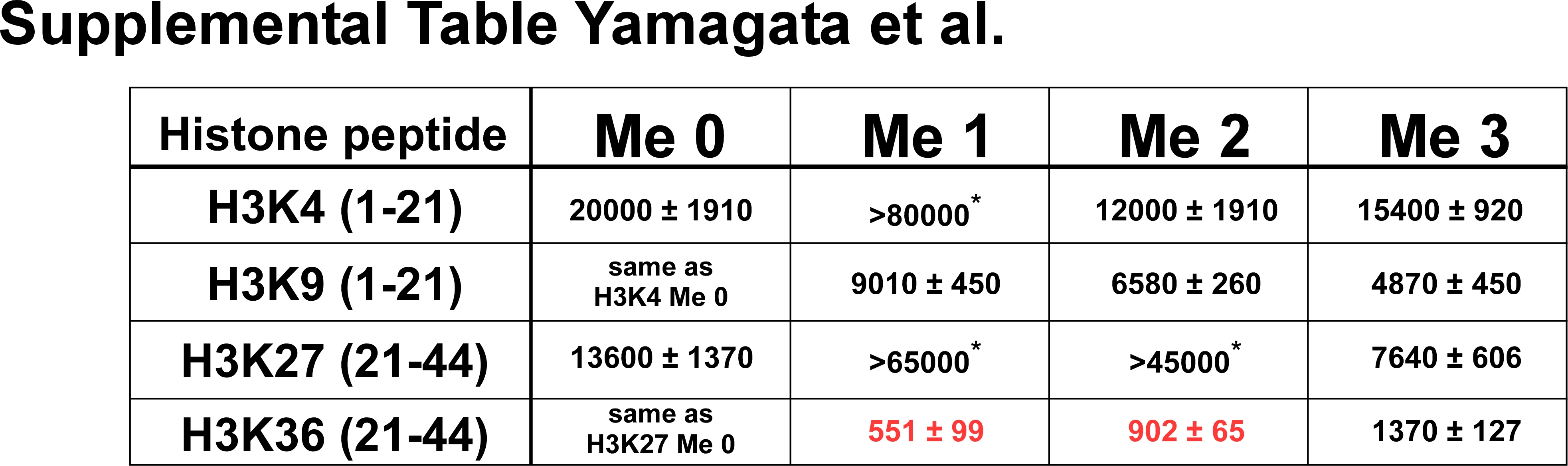

Supplement: Supplementary Data [file mvx004_Supp.zip › jb-17-01-0002-File011.jpg]

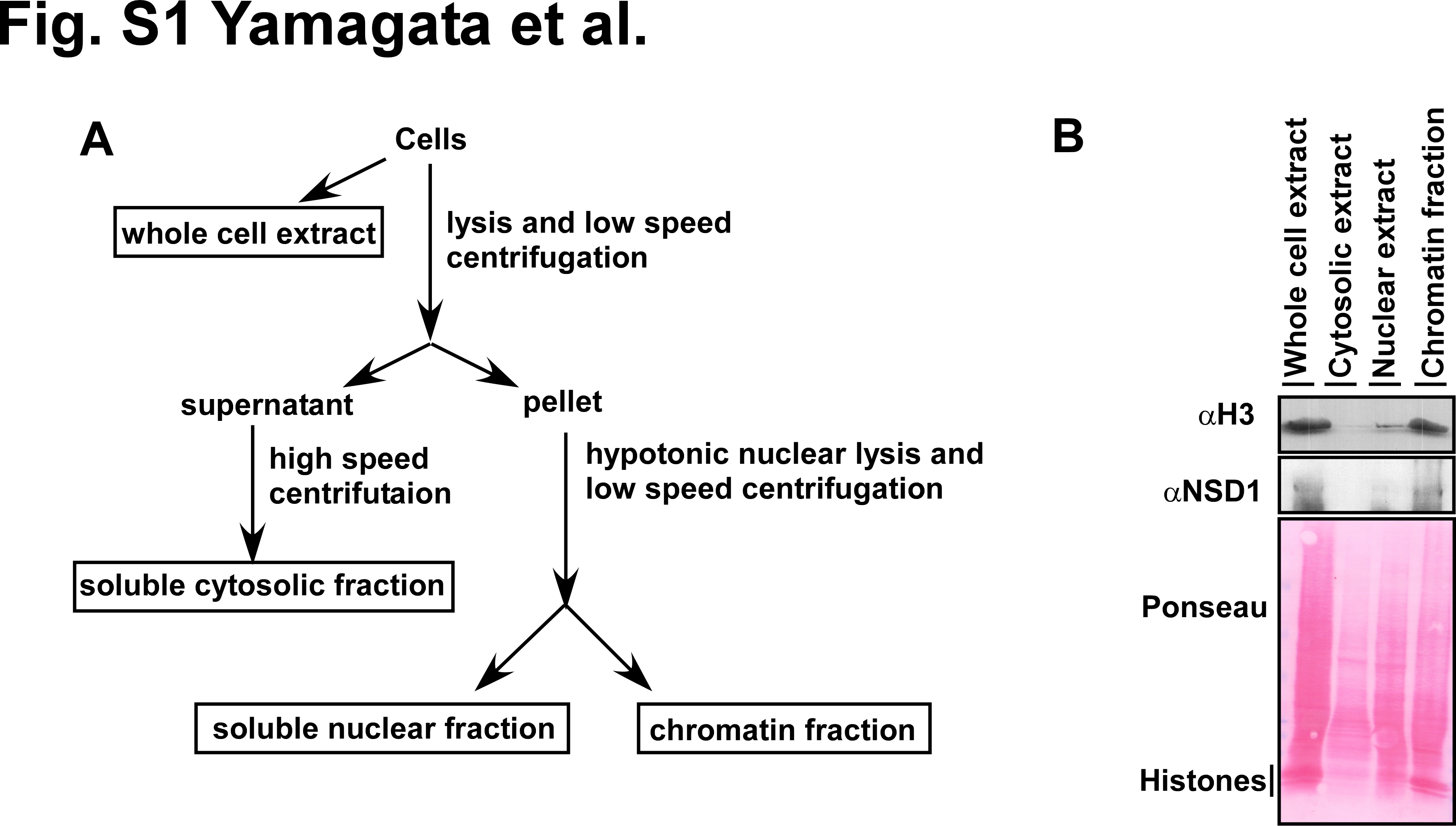

Supplement: Supplementary Data [file mvx004_Supp.zip › jb-17-01-0002-File005.jpg]

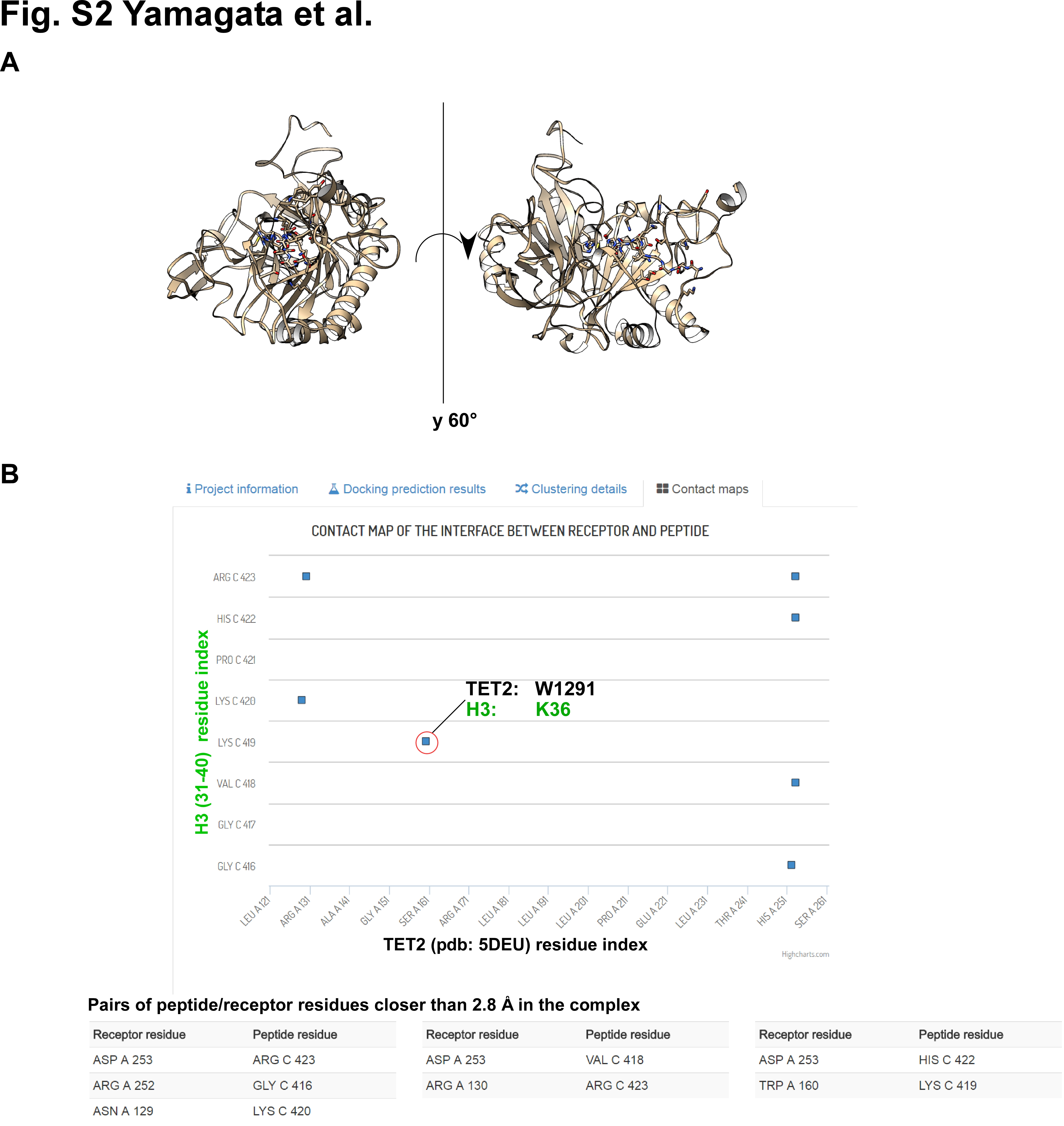

Supplement: Supplementary Data [file mvx004_Supp.zip › jb-17-01-0002-File006.jpg]

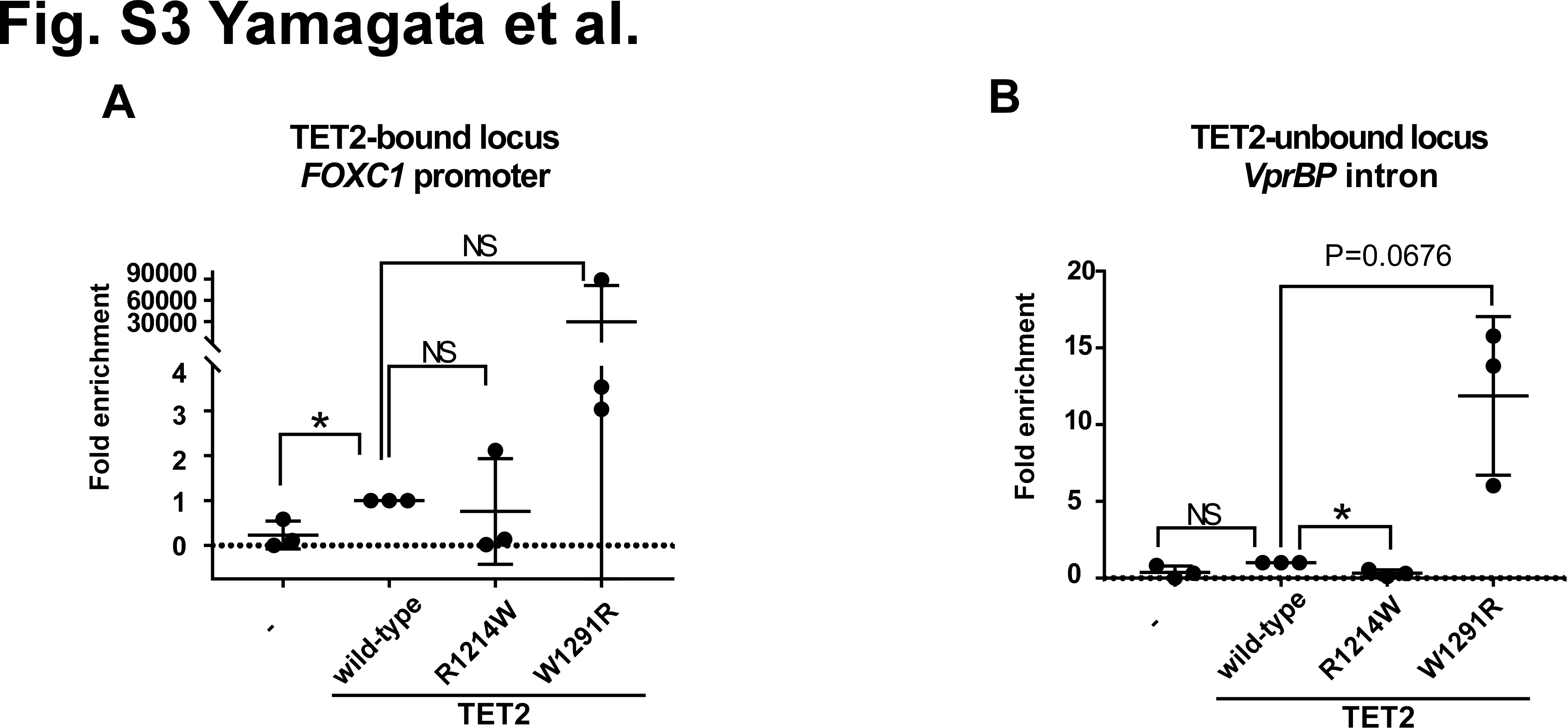

Supplement: Supplementary Data [file mvx004_Supp.zip › jb-17-01-0002-File007.jpg]

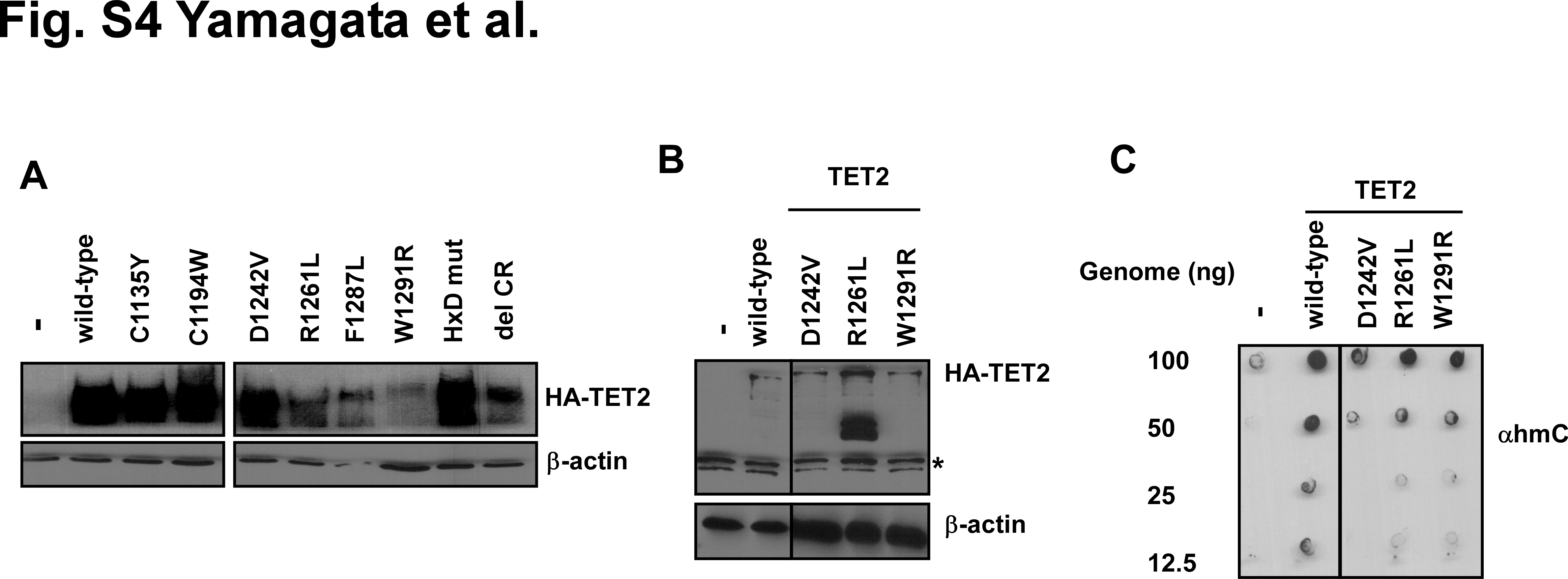

Supplement: Supplementary Data [file mvx004_Supp.zip › jb-17-01-0002-File008.jpg]

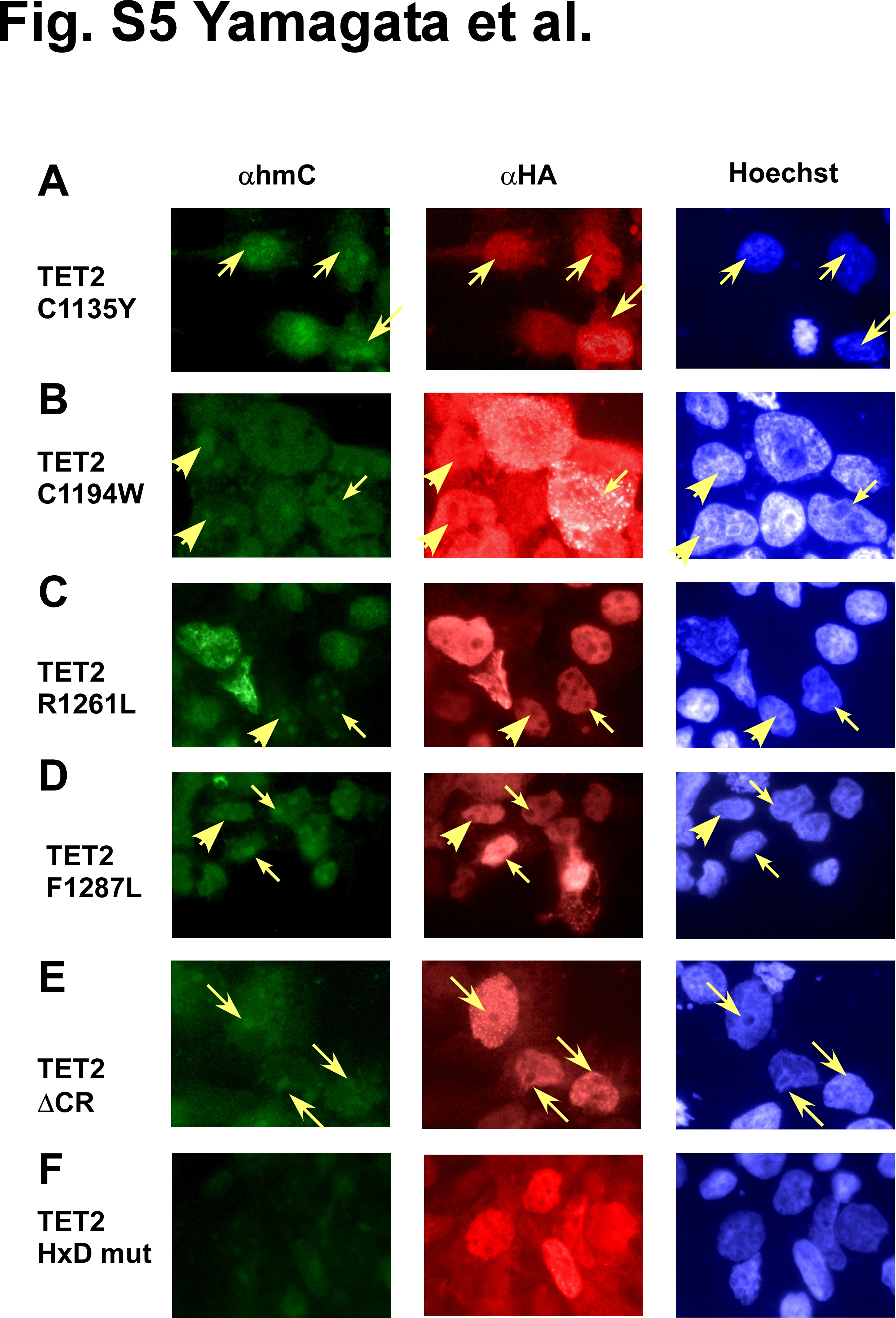

Supplement: Supplementary Data [file mvx004_Supp.zip › jb-17-01-0002-File009.jpg]
