# Supplementary material for: The cysteine-rich domain of TET2 binds preferentially to mono- and dimethylated histone H3K36
Source: J Biochem. 2017 Jan 27;161(4):327–30. doi: 10.1093/jb/mvx004 (PMC5412023; doi:10.1093/jb/mvx004)
Supplement: Supplementary Data [file mvx004_Supp.zip › jb-17-01-0002-File004.doc]

**Supplemental Data**

**The cysteine-rich domain of TET2 binds preferentially to mono- and dimethylated histone H3K36**

Kazuyuki Yamagata and Akira Kobayashi

**Supplemental Experimental Procedures**

**Expression plasmids**

Human TET2 was PCR-amplified from cDNA libraries prepared from HEK293T (RIKEN BRC) cells using Phusion polymerase (NEB) and cloned into the Gateway entry vector pENTR4-D-Topo (Invitrogen). A fully sequenced TET2-WT cDNA, as well as a series of point mutants and a deletion mutant generated by PCR-based mutagenesis, was transferred into the Gateway destination vector pCS2-3HA-DEST (1), which is designed to fuse 3HA-tags to the N-terminus of the protein of interest. The cysteine-rich (CR) domain with its flanking amino acids (residues 1129−1322) was cloned into pGEX4T-1 or pGEX6P-1 (GE Healthcare) using Gibson assembly for expression and purification as GST-fusion proteins in *Escherichia coli*. Point mutations in the CR domain associated with leukaemia patients were introduced by PCR-based targeted mutagenesis. Numbering of amino acid residues is based on GenBank accession numbers NM_001127208.2 and NP_001120680.1 for mRNA and protein, respectively.

**Small-scale biochemical fractionation**

Small-scale biochemical fractionation was performed as previously described (2) with some minor modifications. Briefly, 1 × 10^7^ to 2 × 10^7^ cells were collected, washed with PBS and resuspended at 4 × 10^7^ cells/ml in buffer A (10 mM HEPES, pH 7.9, 10 mM KCl, 1.5 mM MgCl_2_, 0.34 M sucrose, 10% glycerol, 1 mM DTT) containing protease inhibitor cocktail (Roche). Triton X-100 was added to 0.1%, and cells were incubated on ice for 8 min. Nuclei (Fraction P1) were collected by centrifugation (5 min, 1,300 × *g*, 4°C). The supernatant (Fraction S1) was clarified by centrifugation (5 min, 20,000 × *g*, 4°C), and the supernatant (Fraction S2) was collected. P1 nuclei were washed once in buffer A and lysed for 30 min in buffer B (3 mM EDTA, 0.2 mM EGTA, 1 mM DTT and protease inhibitor cocktail), and insoluble chromatin (Fraction P3) was separated by centrifugation (5 min, 1,700 × *g*, 4°C). The P3 fraction was washed once with buffer B and resuspended in SDS sample buffer.

**Protein purification and peptide pull-down assays**

GST-fused CR domains were expressed in *E. coli* and purified using glutathione affinity Sepharose (GE Healthcare). For peptide pull-down experiments, 100 ng of GST-fused CR domains was incubated with 40 ng of biotinylated histone peptide (Anaspec) and incubated in 500 µL binding buffer A (50 mM TRIS-HCl, pH 7.9, at 4°C containing 150 mM NaCl, 0.05% (v/v) NP-40 and 0.1 mM DTT) for 1 h. Protein-peptide complexes were pulled down with glutathione sepharose beads and washed five times with binding buffer A, and bound peptides were detected by western blotting using avidin-HRP (Millipore). Full-length TET2 proteins were expressed in HEK293T cells transfected with wild-type or leukaemia patient-derived TET2 (7 µg per dish with lipofectamine 2000; Invitrogen) for **24 h**. Cells were lysed in binding buffer consisting of 20 mM HEPES, pH 7.9, 150 mM NaCl, 0.05% (v/v) NP-40, 0.1% (v/v) Triton-X, 10% glycerol, 1× protease inhibitor cocktail (Nacalai) and 10 µM Calpeptin (3) (SIGMA), and purified using anti-HA beads (SIGMA) for 4 h at 4°C. Beads were washed three times with binding buffer, and protein-antibody complexes were confirmed by SDS-PAGE followed by CBB staining and used for *in vitro* reactions and binding assays. For the histone peptide pull-down assay, whole-cell extracts from cells expressing TET2 WT or leukaemia-associated mutants were lysed in binding buffer and incubated with 50 ng of biotinylated histone peptide conjugated with 40 µl of Dynabeads M-280 Streptavidin beads (Invitrogen) in 500 µl of binding buffer overnight at 4°C. Protein-peptide complexes were washed five times with binding buffer, and bound 3HA-TET2 proteins were detected by western blotting using anti-HA (3F10; Roche). Nucleosomes mainly composed of mono- and di-units were prepared from HEK293T cells following the conventional protocol (4). Nucleosomes and GST-CR domains were incubated in binding buffer A and pulled down by glutathione sepharose resin, washed with binding buffer A, and analysed by western blotting using pan-H3 Abs (Abcam, ab1791) and anti-GST-HRP (Santa Cruz, sc-459).

**TET2/H3 peptide-binding simulation**

Binding models for TET2 (pdb: 5DEU) with H3 (31−40; ATGGVKKPHR) peptides were simulated using CABS-dock (5) (http://biocomp.chem.uw.edu.pl/CABSdock). The most representative model from the best cluster was selected as the model for fitting. A contact map of the interface between TET2 (5DEU) and H3 (31−40) was calculated by CABS-dock. Pairs of H3/TET2 residues closer than 2.8 Å were analysed on the website, and the model was then confirmed and illustrated using UCSF Chimera software (6) (http://www.rbvi.ucsf.edu/chimera). The H3 peptide is shown in stick representation, and TET2 (5DEU) is shown in ribbon representation.

**Microscale thermophoresis**

Microscale thermophoresis was carried out as described previously (7,8), with some modifications. GST-TET2 CR wild-type or W1291R mutants were labelled with a Monolith NT Protein Labelling Kit following the manufacturer’s instructions (NANOTEMPER technologies). Fluorescence distribution measurements were taken from fluorescently labelled molecules inside a capillary following laser irradiation. Temperature gradients were generated using an IR laser focused on the capillary. Binding affinities were calculating by measuring the temperature jump in the initial stage of irradiation, thermophoretic movement of the molecules within the gradient during the later stages, or both (9,10). Independent experiments were performed for a minimum of three times for each histone modification. Each histone peptide or mononucleosome-binding reaction with CR domains was performed in binding buffer (50 mM TRIS, pH 7.5, 1 M NaCl, 2 mM MgCl_2_, 0.5% Triton X-100) at room temperature for 30 min. Data were plotted using GraphPad Prism software (GraphPad Software).

**Dot-blot analysis**

Cells were incubated with 200 μg/ml proteinase K (Roche) overnight at 55°C, and genomic DNA was isolated with equal volumes of phenol, phenol: chloroform: isoamyl alcohol (25:24:1) and chloroform: isoamyl alcohol (24:1), followed by precipitation with two volumes of ethanol. DNA samples were denatured in 0.4 M NaOH and 10 mM EDTA at 95°C for 10 min and neutralised by adding an equal volume of cold 2 M ammonium acetate (pH 7.0). Two-fold serial dilutions of denatured DNA samples were spotted on a membrane (GE), air-dried, incubated at 80°C for 2 h, and then blocked with 5% non-fat milk for 1 h and incubated with anti-5-hmC antibody (Active Motif; 1:5,000) overnight at 4°C. After incubating with HRP-conjugated anti-rabbit IgG secondary antibody, the membrane was visualised by enhanced chemiluminescence. To ensure equal spotting of total DNA on the membrane, the same blot was stained with 0.02% methylene blue in 0.3 M sodium acetate (pH 5.2; data not shown).

*In vitro* assays of TET2 catalytic activity were performed following the procedures previously described using two different substrates, methylated DNA oligonucleotides (11) and total genomic DNA (12). Genomic DNA from HEK293T cells was isolated using the same method described above.

**Immunofluorescence microscopy**

Staining and observation were carried out as described previously (1). Primary antibodies were used at the following dilutions in permeabilisation buffer: a 1:100 dilution was applied for anti-HA (Santa Cruz, sc-7392) and anti-hmC (Active Motif, 39791); Alexa 594- or Alexa 488-conjugated secondary antibody (Invitrogen) was used at a 1:500 dilution. For localisation of HA-fused TET2 proteins, pCS2-3HA gateway destination vectors carrying HA-TET2 WT and various mutants were used to transfect HEK293T cells with lipofectamine 2000 (Invitrogen). After 24 h, cells were subjected to immunofluorescence using anti-HA mAb as described above.

**Confocal fluorescence microscope imaging**

Hela cells were transfected with HA-TET2 WT and various mutants using lipofectamine 2000 (Invitrogen), and subjected to immunofluorescence after **24 h** using rat anti-HA (3F10; Roche; 1:1000 dilution) and anti-H3K36me2 mAb (13) (1:1000 dilution) following immunofluorescence microscopy sectioning. Cells were imaged using a confocal fluorescence microscope (LSM710; Carl Zeiss) and analysed by ZEN software (blue edition; Carl Zeiss). For line profiles, images were analysed by ZEN, and the intensity of each signal was graphed using Microsoft Excel.

**Chromatin immunoprecipitation (ChIP)**

Chromatin immunoprecipitation (ChIP) was carried out as described previously (14) with some modifications. HEK293T cells (1 × 10^7^) were plated in a 10 cm dish. At 70−80% confluency, cells were transfected for 24 h with 20 µg of 3× HA-tagged constructs using lipofectamine 2000. Cells were crosslinked with 1% methanol-free formaldehyde (Pierce) for 10 min at 37°C, quenched with 0.125 M glycine for 5 min at room temperature and washed three times with ice-cold PBS. Cells were incubated with Swelling buffer (25 mM HEPES, pH 7.9, 1.5 mM MgCl_2_, 10 mM KCl, 0.1% NP-40, protease inhibitor cocktail and **10 µM Calpeptin**) on ice for 10 min, and then scraped and collected by centrifugation (5000 × *g*, 4°C, 5 min). Pellets were resuspended in sonication buffer (50 mM HEPES, pH 7.9, 140 mM NaCl, 1 mM EDTA, 1% Triton X-100, 0.1% Na-deoxycholate, 0.1% SDS, protease inhibitor cocktail, **10 µM Calpeptin**; 1 × 10^7^ cells/ml) and sonicated using a Bioruptor (CosmoBioJapan) in cold water using 10 cycles of 5 min at high sonication strength with 30 s pulses. Sonicated chromatin was clarified by centrifugation twice at 16,000 × *g* for 5 min then pre-cleared with 20 µl of protein G-magnetic beads (Dynabeads, Invitrogen) for 2 h at 4°C with rotation. Supernatants were collected using a magnetic stand (Promega) and immunoprecipitated with anti-HA (5 μg 12CA5, Roche) or anti-H3K36me2 (5 µg) (13) for 24 h at 4°C on a rotating wheel. Magnetic beads were washed for 5 min at 4°C with 1 ml of each of the following wash buffers: once with sonication buffer, once with wash buffer A (50 mM HEPES, pH 7.9, 500 mM NaCl, 1 mM EDTA, 1% Triton X-100, 0.1% Na-deoxycholate and 0.1% SDS), once with wash buffer B (20 mM TRIS-HCl, pH 8.0, 250 mM LiCl, 0.5% NP-40 and 0.5% Na-deoxycholate) and twice with TE buffer. To reverse the crosslinking and release the DNA, beads were added to freshly prepared elution buffer (50 mM TRIS-HCl, pH 7.5, 1 mM EDTA, 1% SDS) and incubated at 65°C for 5 min, and then mixed at RT at 1,200 rpm for 15 min. Isolated DNA fragments were incubated with proteinase K (Wako) at 45°C for 2 h and recovered using a PCR clean-up kit (Favorgen). DNA was then quantified by fluorimetry using Quantus (Promega), and 1 ng of enriched fragmented DNA or input was subjected to 40 cycles of quantitative PCR using a Thermal Cycler Dice Real-time System II (TAKARA). Primers for ChIP-qPCR were as follows:

HOXA4 F, TCCTCGAAGGGAGGGAACTT;

HOXA4 R, CTCGGCAAGCCCAGAAAAA;

FOXC1 F, CCTGTGAGCCAGATGCTGAA;

FOXC1 R, CCCTTTCCAACCCGCAGTAT;

RPL30 F, AACTGCCCAGCTTTGAGGTAATCG;

RPL30 R, ATGAAGGGCCAAACACCACAATCG;

VprBP F, AAGCCCGTACGGAACAGAGA;

VprBP R, GGAGGGGCGCATTTCATTTC.

**SUPPLEMENTAL FIGURE LEGENDS**

**Figure S1. Fractionation of cellular proteins** (related to Fig. 1B).

(A) Biochemical fractionation scheme. (B) HEK293T cells were fractionated following the scheme in Fig. S1A, and the same amount of extract was subjected to immunoblotting with anti-H3 and anti-NSD1. Protein levels were also confirmed by Ponceau staining.

**Figure S2. Binding simulation of TET2 (pdb: 5DEU) to an H3 peptide (residues 31−40) calculated by CABS-dock** (related to Fig. 1F).

(A) Overview of the binding simulation of TET2 (5DEU) with the H3 peptide (residues 31−40) calculated by CABS-dock in two different views. The H3 peptide is shown in stick representation, and TET2 (pbd: 5DEU) is shown in ribbon representation. (B) Contact map of the interface between TET2 (5DEU) and H3 (31−40). Pairs of H3/TET2 residues closer than 2.8 Å are shown in the bottom column. The pdb file of this model is available at JB online.

**Figure S3. CR mutations in TET2 disrupt the correct recruitment to its target and non-target loci** (related to Fig. 2E and F).

(A and B) ChIP-qPCR performed on TET2-transfected HEK293T cells. *P<0.05 (student t-test).

**Figure S4. TET2 mutations in the CR domain alter enzymatic activity in cells when expressed at the same level** (related to Fig.2G).

(A) Expression levels of HA-TET2 proteins were confirmed by western blotting. β-actin was used as a loading control. (B and C) CR mutations in TET2 alter enzymatic activity in cells when expressed at the same level. Dot-blot analysis of 5-hmC levels in HEK293T cells over-expressing wild-type or mutant TET2 cDNA (the same amount in each case).

**Figure S5. TET2 mutations in the CR domain affect both enzymatic activity and cellular localisation *in vivo*** (related to Fig. 2H–K).

(A–D) Immunocytochemical detection of 5-hmC (green) and HA-TET2 (red) in HEK293T cells transiently transfected with mutant TET2 cDNA.

**Figure S6. Graphical abstract of current work.**

The CR domain of TET family proteins is a nucleosome-binding module that binds preferentially to H3K36me1/2. CR mutations in leukaemia patients disrupt the recognition of H3K36 methylation, which alters enzymatic activity of TET2 in cells.

**Supplementary table. The cysteine-rich domain of TET2 binds preferentially to mono- and dimethylated histone H3K36.**

Experimental microscale thermophoresis-derived binding affinities for binding between histone H3 peptides methylated at various lysine residues and GST-TET2 CR wild-type. Kd values were determined by Monolith NT.115 and subsequently analysed by PRISM software. Kd represents nM. The asterisk indicates that Kd was not accurately determined due to low affinities. The Kd of H3K79 peptides could not be determined because their peptides formed aggregation during measurement.

**Supplemental References**

1.	Iwase, S., Xiang, B., Ghosh, S., Ren, T., Lewis, P. W., Cochrane, J. C., Allis, C. D., Picketts, D. J., Patel, D. J., Li, H., and Shi, Y. ATRX ADD domain links an atypical histone methylation recognition mechanism to human mental-retardation syndrome. *Nat Struct Mol Biol* **18**, 769-776

2.	Wysocka, J., Reilly, P. T., and Herr, W. (2001) Loss of HCF-1-chromatin association precedes temperature-induced growth arrest of tsBN67 cells. *Mol Cell Biol* **21**, 3820-3829

3.	Wang, Y., and Zhang, Y. (2014) Regulation of TET protein stability by calpains. *Cell Rep* **6**, 278-284

4.	Dai, B., Dahmani, F., Cichocki, J. A., Swanson, L. C., and Rasmussen, T. P. (2011) Detection of post-translational modifications on native intact nucleosomes by ELISA. *J Vis Exp*

5.	Kurcinski, M., Jamroz, M., Blaszczyk, M., Kolinski, A., and Kmiecik, S. (2015) CABS-dock web server for the flexible docking of peptides to proteins without prior knowledge of the binding site. *Nucleic Acids Res* **43**, W419-424

6.	Pettersen, E. F., Goddard, T. D., Huang, C. C., Couch, G. S., Greenblatt, D. M., Meng, E. C., and Ferrin, T. E. (2004) UCSF Chimera--a visualization system for exploratory research and analysis. *J Comput Chem* **25**, 1605-1612

7.	Alpatov, R., Lesch, B. J., Nakamoto-Kinoshita, M., Blanco, A., Chen, S., Stutzer, A., Armache, K. J., Simon, M. D., Xu, C., Ali, M., Murn, J., Prisic, S., Kutateladze, T. G., Vakoc, C. R., Min, J., Kingston, R. E., Fischle, W., Warren, S. T., Page, D. C., and Shi, Y. (2014) A chromatin-dependent role of the fragile X mental retardation protein FMRP in the DNA damage response. *Cell* **157**, 869-881

8.	Greer, E. L., Beese-Sims, S. E., Brookes, E., Spadafora, R., Zhu, Y., Rothbart, S. B., Aristizabal-Corrales, D., Chen, S., Badeaux, A. I., Jin, Q., Wang, W., Strahl, B. D., Colaiacovo, M. P., and Shi, Y. (2014) A histone methylation network regulates transgenerational epigenetic memory in C. elegans. *Cell Rep* **7**, 113-126

9.	Wienken, C. J., Baaske, P., Rothbauer, U., Braun, D., and Duhr, S. (2010) Protein-binding assays in biological liquids using microscale thermophoresis. *Nat Commun* **1**, 100

10.	Jerabek-Willemsen, M., Wienken, C. J., Braun, D., Baaske, P., and Duhr, S. (2011) Molecular interaction studies using microscale thermophoresis. *Assay Drug Dev Technol* **9**, 342-353

11.	Tahiliani, M., Koh, K. P., Shen, Y., Pastor, W. A., Bandukwala, H., Brudno, Y., Agarwal, S., Iyer, L. M., Liu, D. R., Aravind, L., and Rao, A. (2009) Conversion of 5-methylcytosine to 5-hydroxymethylcytosine in mammalian DNA by MLL partner TET1. *Science* **324**, 930-935

12.	Hu, L., Li, Z., Cheng, J., Rao, Q., Gong, W., Liu, M., Shi, Y. G., Zhu, J., Wang, P., and Xu, Y. (2013) Crystal structure of TET2-DNA complex: insight into TET-mediated 5mC oxidation. *Cell* **155**, 1545-1555

13.	Kimura, H., Hayashi-Takanaka, Y., Goto, Y., Takizawa, N., and Nozaki, N. (2008) The organization of histone H3 modifications as revealed by a panel of specific monoclonal antibodies. *Cell Struct Funct* **33**, 61-73

14.	Brookes, E., de Santiago, I., Hebenstreit, D., Morris, K. J., Carroll, T., Xie, S. Q., Stock, J. K., Heidemann, M., Eick, D., Nozaki, N., Kimura, H., Ragoussis, J., Teichmann, S. A., and Pombo, A. (2012) Polycomb associates genome-wide with a specific RNA polymerase II variant, and regulates metabolic genes in ESCs. *Cell Stem Cell* **10**, 157-170
